# Posterior reversible encephalopathy syndrome (PRES) in children – a summary of 20 years of tertiary neurology centre experience

**DOI:** 10.3389/fneur.2026.1944274

**Published:** 2026-09-11

**Authors:** Nadja Bednarczuk, Evangelia Ioannidou, Kshitij Mankad, Sandeep Prasad, Steward G. Boyd, Marios Kaliakatsos, Sheba Azam, Kitty Howse, Nivedita Desai

**Affiliations:** 1 Department of Neurology, Great Ormond Street Hospital, London, United Kingdom; 2 University College London Great Ormond Street Institute of Child Health, London, United Kingdom; 3 Department of Radiology, Great Ormond Street Hospital for Children, London, United Kingdom; 4 Department of Neurophysiology, Great Ormond Street Hospital for Children, London, United Kingdom

**Keywords:** clinical management, diagnosis, long-term outcomes, neurophysiology, neuroradiology, posterior reversible encephalopathy syndrome (PRES)

## Abstract

**Objective:**

Posterior reversible encephalopathy syndrome (PRES) is a clinico-radiological syndrome characterised by seizures, hypertension, visual disturbances alongside classical parieto-occipital lesions on neuroimaging. We report the clinical presentation and management of the largest UK paediatric PRES cohort.

**Methods:**

Seventy-seven children (x̄ age = 8.6 years [SD = 4.3], 64% male) experienced PRES as inpatients at Great Ormond Street Hospital (London, UK) from January 2005 to May 2024. Electronic patient records were retrospectively analysed.

**Results:**

Most children (97.4%) had significant co-morbidities, including haem-oncological (*n* = 25, 32.5%), renal (*n* = 17, 22.1%) and immunological conditions (*n* = 9, 11.7%). Fifty-four patients (70.1%) took immunomodulating medications. Seizures were the commonest presenting symptom (*n* = 60, 77.9%), followed by headaches (*n* = 15, 19.4%), visual disturbance (*n* = 13, 16.8%) and encephalopathy (*n* = 18, 23.3%). Fifty (64.9%) children were hypertensive. Anti-seizure medication (ASM) was commenced in fifty-six children (72.7%), with Levetiracetam (*n* = 36; 64.3%) being most used. Thirty-six children (64.3%) required short-term (<6 months) ASMs and twenty (35.7%) remained on long-term treatment (>6 months). Indications for long-term ASMs included persistent seizures (*n* = 12) or neurological symptoms (*n* = 3) and underlying co-morbidities (*n* = 2). Neuroimaging (MRI *n* = 70 (90.9%); CT *n* = 7(9.1%)) was performed in all patients. Twenty-four (*n* = 24) had the typical parieto-occipital pattern of involvement, whilst forty (*n* = 40) children had atypical patterns. Seventy-four (*n* = 74, 96%) patients underwent EEG; sixty-seven of these (*n* = 67, 90.5%) had abnormal EEG findings. Focal slowing showed a trend towards long-term ASM-use (aOR = 1.49, 95% CI 0.28–7.88; *p* = 0.08).

**Conclusion:**

Our cohort showcases the broad clinical spectrum of paediatric PRES. Global standardised clinical pathways are needed to better inform both acute and long-term management and outcomes.

## Introduction

Posterior Reversible Encephalopathy Syndrome (PRES) is a clinico-radiological condition characterised by variable combinations of seizures, altered consciousness, headaches, and visual disturbances (1). It is also associated with hypertension. Distinctive neuroimaging findings, affecting the parieto-occipital regions of the brain, are also frequently noted (2). Since its initial description by Hinchey et al. in 1996 (3), PRES has been increasingly recognised in both adult and paediatric populations, yet its exact pathophysiology remains elusive (4). The prevailing theories suggest endothelial dysfunction, cerebral autoregulatory failure, and immune-mediated injury as primary mechanisms leading to vasogenic oedema and, consequently, the aforementioned neurological symptoms (5). While traditionally considered a transient and reversible syndrome, recent studies indicate that atypical presentations, recurrent episodes, and long-term neurological sequelae may complicate its course (6, 7). However, prognostic data remains limited.

Paediatric PRES is a rare condition, with an estimated incidence of 0.04% (8) and only 449 cases reported across 272 published studies in non-oncological patients (9). Indeed, children with malignancies are more commonly affected, with reported incidences of up to 0.7% (10, 11). Other complex co-morbidities, such as renal failure and post-transplantation immunosuppression, also increase the likelihood of PRES (Cite: 35801219).

We present the largest known UK paediatric PRES cohort from a tertiary neurology centre, reviewing 20 years of clinical experience at Great Ormond Street Hospital (GOSH). Our objective is to characterise the clinical features of paediatric PRES, including neuroimaging and EEG data, analyse treatment patterns, and evaluate outcomes, including the need for long-term anti-seizure medications (ASM). Hereby, we aim to increase the modest evidence-base guiding our understanding of current management practices. By integrating clinical presentation, imaging abnormalities, and EEG data, we aim to identify potential predictors of outcomes in paediatric PRES. Given the heterogeneity in presentation and the variation in standardised management protocols, we also aim to highlight gaps in current clinical pathways and share clinical experience in diagnostic and therapeutic strategies.

## Methods

This service evaluation comprised data from Great Ormond Street Hospital (GOSH), a tertiary paediatric neurology centre in London, UK over twenty years (January 2005 to May 2024). We retrospectively evaluated the clinical characteristics, imaging findings, EEG studies, management, and outcomes of paediatric patients with Posterior Reversible Encephalopathy Syndrome (PRES). The project was registered with the GOSH Clinical Audit and Service Evaluation Department (Registration No. 3474). As a registered service evaluation using routinely collected anonymised clinical data, formal NHS Research Ethics Committee (REC) approval was not required. Data collection complied with institutional information governance policies and the UK General Data Protection Regulation (GDPR).

**Figure 1:**
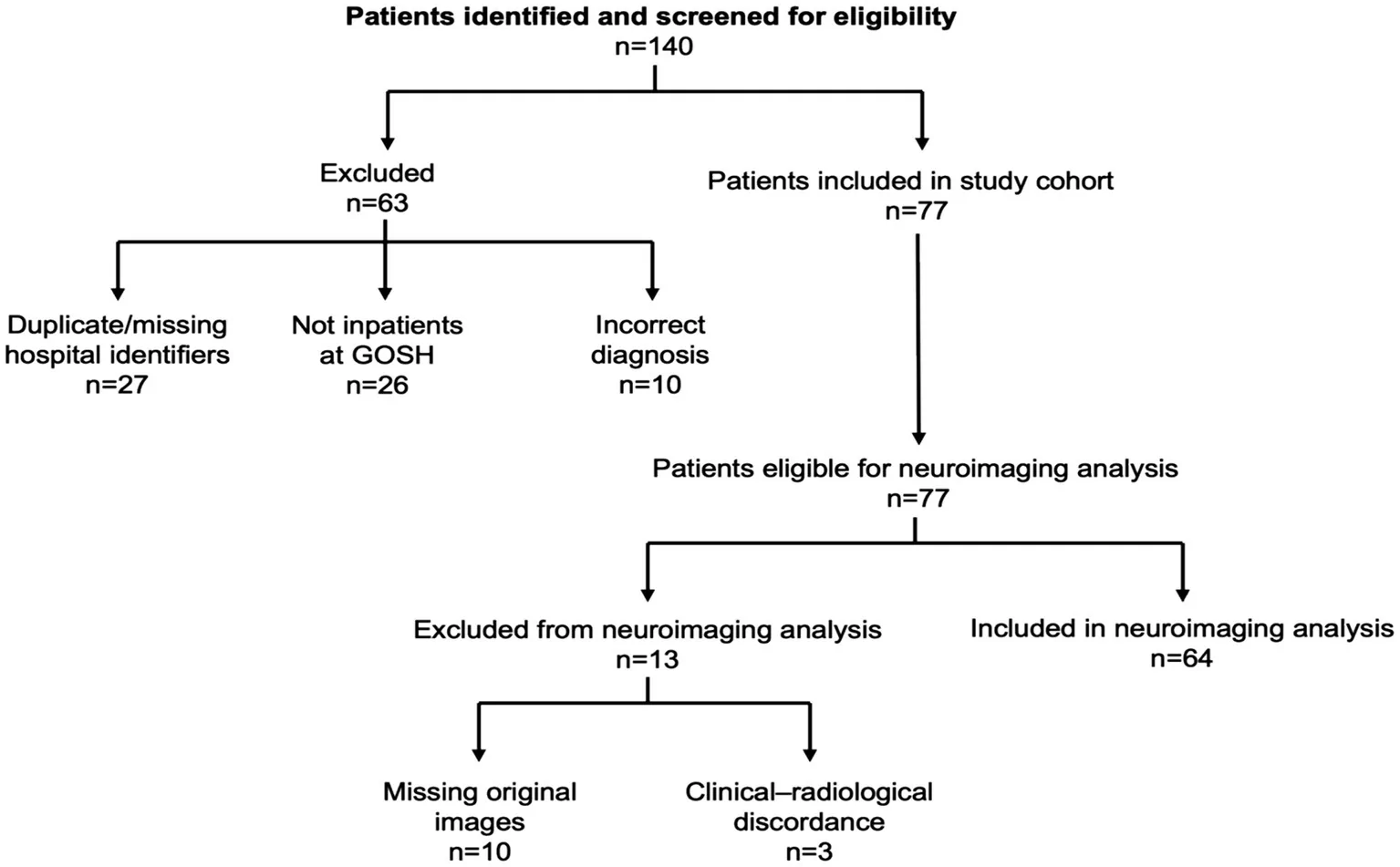
Patient selection flow chart. Flow chart illustrating patient selection. One-hundred-forty patients were screened for eligibility, with 77 included in the final study cohort. A further 13 patients were excluded from detailed neuroimaging analysis due to clinical-radiological discordance (*n* = 3) and missing original images (*n* = 10).

Patients were identified via electronic health records and clinical and neuroradiology databases. A total of 140 patient records were screened by two independent reviewers (NB, EI). Exclusion criteria included patients who were not inpatients at GOSH at the time of their PRES episode, unconfirmed cases, duplicate records, and entries with missing or incorrect hospital identifiers. The final study cohort comprised 77 patients with confirmed PRES diagnoses during their inpatient stay at GOSH (Figure 1).

PRES diagnoses were made as part of the clinical care provided at time of the patient’s admission. This included specialist neurological assessment and ongoing review, independent neuroradiological review of imaging, and multi-disciplinary discussion of each case to confirm diagnosis. On retrospective review of cases, ND, NB, and EI independently reviewed cases to confirm documented clinical diagnosis. KM and SP independently assessed available imaging and imaging reports, applying standardised neuroradiological criteria. PRES diagnosis was finalised by combining compatible clinical features with neuroimaging findings. Any unconfirmed cases were excluded. Cases with atypical imaging patterns were reviewed by ND, KM, and ND to confirm that clinical findings, patient trajectory and imaging remained compatible with PRES and alternative diagnoses did not better explain the radiological findings.

A standardised data collection proforma was applied. Demographic information included age at diagnosis, sex, and underlying co-morbidities. Clinical presentation was assessed, with a focus on symptoms at onset, including seizures, headaches, altered mental status, visual disturbances, and hypertension. Relevant clinical variables were determined using symptoms typical of PRES reported in the evidence base (1). Hypertension was defined clinically by the attending clinician due to persistent systolic or diastolic blood pressures above the 95th centile for the child’s age, sex, and height (12). Investigations such as neuroimaging (MRI, CT) and electroencephalography (EEG). Management strategies were noted, including use of antihypertensives and anti-seizure medication (ASM), and the need for paediatric neurology follow-up. Long-term outcomes including long-term ASM use (i.e., > 6 months of treatment), and persistent neurological symptoms (i.e., any persistent neurological symptom or sign reported at outpatient follow-up after resolution of PRES episode). Long-term ASM use did not confer a diagnosis of epilepsy. Recurrent PRES was defined as a distinct, second episode of PRES confirmed both clinically and radiologically in the same patient. As a tertiary paediatric neuroscience centre, EEG was readily available throughout the study period and was performed according to clinical need; therefore, the level of EEG access and utilisation in our cohort may not reflect routine practice in other healthcare settings.

All children who underwent MRI were scanned using a routine protocol that included pre- and post-contrast sequences. The protocol comprised standard T1-weighted imaging (T1WI), diffusion-weighted imaging (DWI), susceptibility-weighted imaging (SWI), T2-weighted imaging (T2WI), fluid-attenuated inversion recovery (FLAIR), and post-contrast T1-weighted sequences. GOSH introduced 3 T MRI in 2015; before this, imaging was performed predominantly using 1.5 T scanners. Our 2005–2024 cohort therefore includes patients investigated in both MRI eras. A sensitivity analysis comparing differences in outcomes including PICU admissions, EEG abnormalities, imaging findings of follow-up gliosis, and ASM treatment durations between these two study periods (2005–2014 versus 2015–2024) was performed, and found no significant differences (Table S1). MRI and CT scans of 77 children with clinical signs of PRES (2, 13) were analysed. Detailed retrospective neuroradiological review was possible in 64 children (61 MRI and three CT). In the remaining 13 cases, the original diagnostic imaging was unavailable for direct retrospective review in ten cases, reflecting changes in electronic and radiological archiving systems over the 20-year study period. In three cases, there was clinical-radiological discordance, with imaging unable to confirm a diagnosis but documented clinical features and management in keeping with PRES (Figure 1). It is important to note that absence of the original imaging for specialist neuroradiology retrospective review did not equate to absence of radiological evidence at the time of diagnosis; these patients had been identified through detailed clinical and radiological documentation of PRES and reports showcasing that are available. A total sample size of 64 was therefore obtained for detailed neuroimaging review, out of which 61 children had MRI scans while three only had CT head done. Sensitivity analyses showed no significant differences in significance directions for models assessing clinical outcomes (e.g., PICU admissions, ASM duration) when comparing the entire study cohort (*n* = 77) to those included within the neuroimaging cohort (*n* = 64) (Table S2).

### Statistical analysis

Data were analysed in R (Version R 3.6.0+). Descriptive statistics were employed to summarise demographic and clinical characteristics. Outcome variables were chosen *a priori* based on clinical relevance and evidence from published literature. Continuous variables were assessed for normality using the Shapiro-Wilks test and expressed as means with standard deviations or medians with interquartile ranges, depending on the data distribution, and compared with t-tests using Bonferonni corrections. Categorical variables were presented as frequencies and percentages and compared using chi-squared analysis. Associations between the binary outcome variables of PICU admission and ASM duration (short vs. long-term) and predictor variables were assessed with bias-reduced logistic regressions using the *brglm2* R package (14). This method was utilised to reduce small-sample size bias in subgroup analysis and address potential separation in the data, which can produce inaccurate, unstable odds estimates in conventional logistic regression. By applying bias-reducing adjustments to the standard estimation equations of conventional logistic regression, this analysis produces more accurate odds estimates in smaller sample sizes. Predictor variables for PICU admission included children’s presenting signs (Hypertension, Seizures, headache, encephalopathy, vision changes). Factors affecting ASM duration included the presence of focal slowing on EEG. All models were adjusted for age. Adjusted odds ratios (aOR), 95% confidence intervals (CI) and two-sided *p*-values were reported for all bias-reduced models.

## Results

### Demographics and past medical history

**Figure 2:**
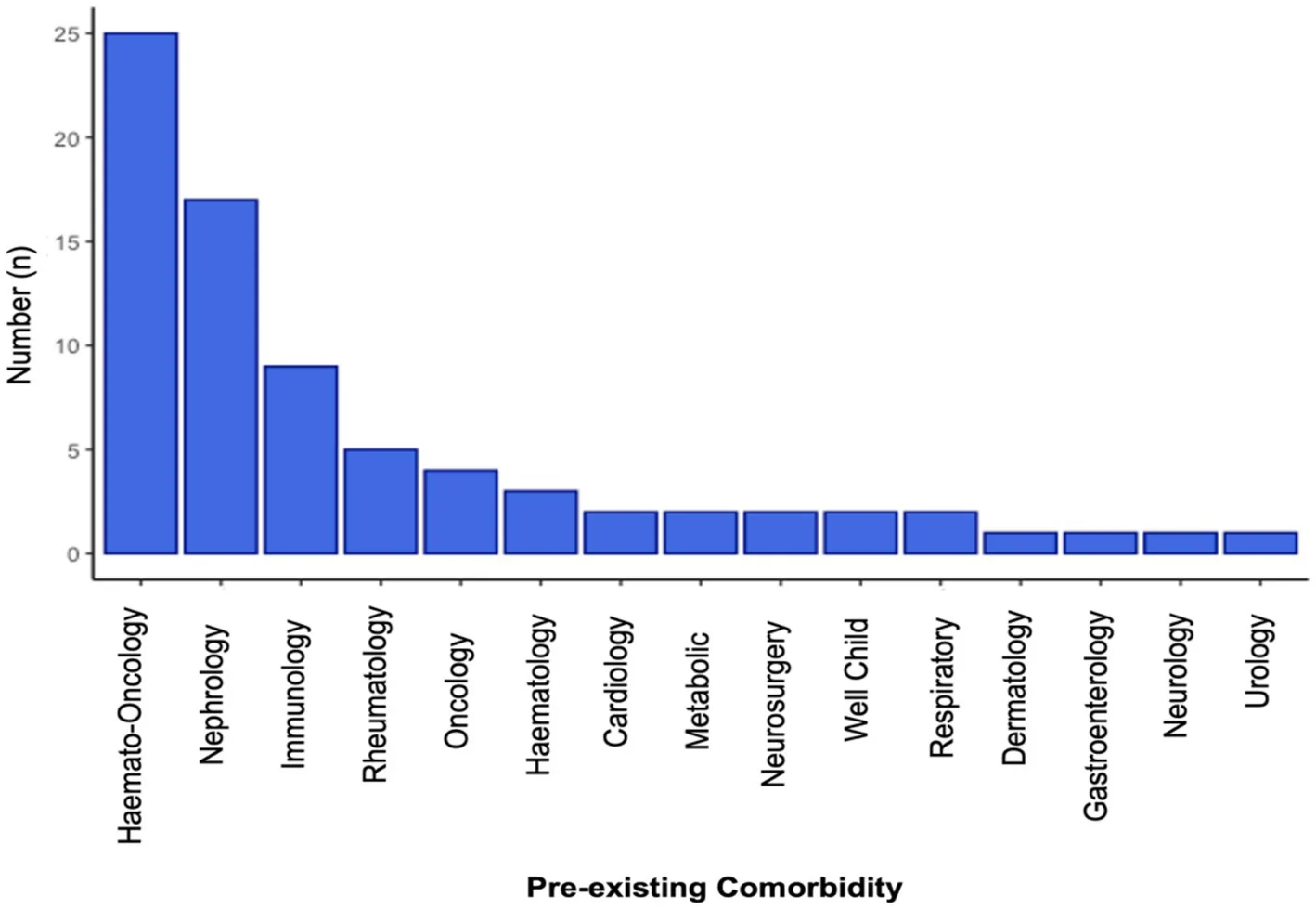
Categories of past medical history amongst PRES patients. Bar chart depicting the most frequent categories of pre-existing medical conditions in our cohort of patients with PRES, with the number of affected patients (*n*) represented on the *y*-axis. Haematological/oncological (*n* = 25, 32.5%) alongside renal (*n* = 17, 22.1%) and immunological conditions (*n* = 9, 11.7%) being the most prevalent.

Seventy-seven children, with a mean age of 8.6 years (Range = 6 months – 16 years, SD = 4.3 years) with a slight male predominance (*n* = 49, 64%) experienced PRES whilst inpatients at GOSH. Most (*n* = 75, 97.4%) had underlying co-morbidities. Haem-oncological (*n* = 25, 32.5%) alongside renal (*n* = 17, 22.1%) and immunological conditions (*n* = 9, 11.7%) were the most common (Figure 1). Due to their underlying conditions, many patients were taking medications that have been linked to the development of PRES. Fifty-four patients (70.1%) took immunomodulating drugs such as ciclosporin (*n* = 26; 48.1%), tacrolimus (*n* = 8, 14.8%), methotrexate (*n* = 6, 11.1%) and systemic steroid medication (*n* = 17, 31.5%). Nine children were receiving chemotherapy (Figure 2).

The patient with underlying neurological co-morbidities experienced symptoms of Guillain-Barre Syndrome and has previously been reported in the literature (15). Two patients had no significant past medical history. One of these children had an intercurrent streptococcal infection, however no other systemic sequalae of streptococcal infection, whilst the other had no evidence of intercurrent illness or other predisposing factors. A total of nine children (11.6%) had intercurrent infections, with a range of bacterial (e.g., enterococcus, streptococcus) and viral pathogens (e.g., influenza, varicella) confirmed on microbiological testing.

### PRES presentation

**Figure 3:**
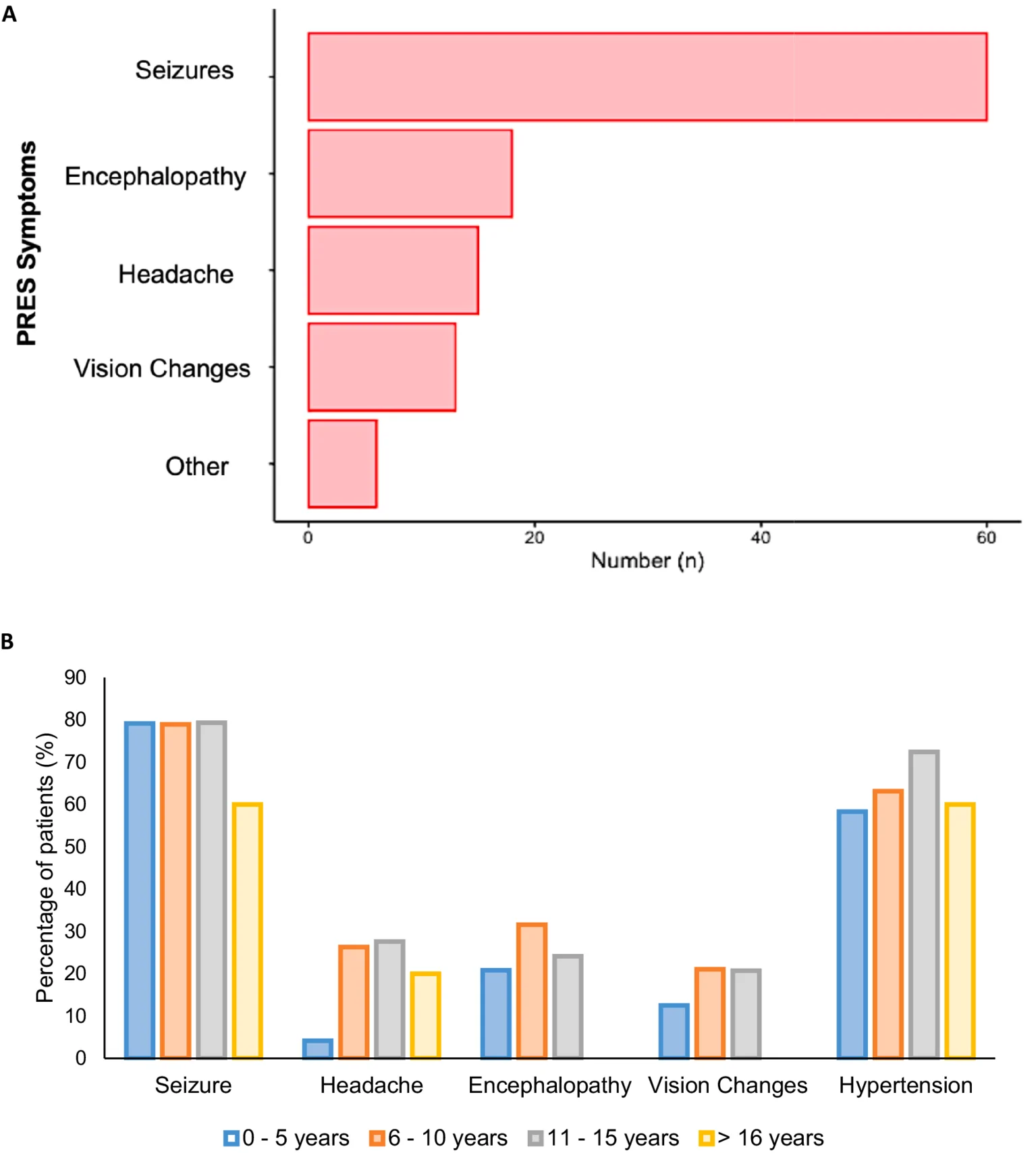
PRES presentation. **(A)** Bar chart showing the most frequent presenting symptoms of PRES at time of diagnosis, with the number of affected patients (*n*) represented on the x-axis. Seizures were the commonest presenting symptom (*n* = 60, 77.9%). Headaches (*n* = 15, 19.4%), visual disturbance (*n* = 13, 16.8%), and encephalopathy (*n* = 18, 23.3%) were also reported. **(B)** Differences in PRES presenting symptoms across different age groups, with the percentage of affected patients depicted on the y-axis. No difference in presenting symptoms were observed. Of note, headaches were less commonly noted in children aged 0–5 years, likely due to difficulty in describing subjective symptoms.

Seizures were the commonest presenting symptom (*n* = 60, 77.9%), typically generalised tonic–clonic seizures. Headaches (*n* = 15, 19.4%), visual disturbance (*n* = 13, 16.8%) and encephalopathy (*n* = 18, 23.3%) were also reported (Figure 3A). Other symptoms included behavioural changes, motor function disturbances (e.g., hemiplegia, focal deficits) and slurred speech (Figure 3B). Fifty patients (65%) were hypertensive. Hypertensive children were more likely to experience headaches (*n* = 15, χ2 = 5.62, *p* = 0.017), but not more likely to have seizures (*n* = 39, χ2 = 0.55, *p* = 0.45) or be encephalopathic (*n* = 14, χ2 = 0.54, *p* = 0.46).

Twenty children (25.9%) required intensive care admission. There was no age difference in those children requiring PICU admission (mean age = 9.4 years) and those that did not (mean age = 8.5 years; *p* = 0.453). Most children requiring intensive care were hypertensive (75%, *n* = 15, χ2 = 0.00054, *p* = 0.96) and experienced seizures (85%, *n* = 17, χ2 = 0.33, *p* = 0.56). Bias-reduced logistic regression adjusted for age and underlying disease demonstrated that presenting symptoms and signs, including the presence of hypertension, headache, encephalopathy, seizures, or vision changes, did not independently predict the need for PICU admission (likelihood ratio χ^2^ (12) = 8.73, *p* ≈ 0.72; AIC = 100.35). Hypertension had the highest estimated odds of the outcome (aOR = 1.69, 95% CI 0.42–6.71, *p* = 0.46), but this did not reach statistical significance.

### Patient management

Anti-hypertensive medication was started in all children with raised blood pressures. The most utilised agent was Amlodipine (*n* = 26, 52%), followed by beta blockers (e.g., Atenolol, Labetalol; *n* = 13, 26%). Twenty-one children (42%) required more than one anti-hypertensive to lower their blood pressure. Of those children on immunomodulating medication (*n* = 54), thirty-one (57.4%) had changes made to their background medication including stopping the potential contributing medication or changing to a different immunomodulating drug.

Most children (*n* = 56, 72.7%) were commenced on regular anti-seizure medications (ASM), of which 36 (64.3%) were prescribed Levetiracetam (*n* = 36; 64.3%). Less commonly utilised ASMs included phenytoin (*n* = 9, 16.1%), Clobazam (*n* = 4, 7.1%), Sodium Valproate (*n* = 2, 3.6%), and Phenobarbitone (*n* = 1, 1.8%). Thirty-six children (64.3%) required only short-term (i.e., <6 months) ASMs, whilst twenty (35.7%) remained on long-term treatment for more than 6 months.

Indications for long-term ASMs included persistent seizures post-PRES (*n* = 12, 60%) or neurological symptoms unrelated to PRES (*n* = 3, 15%), and associated co-morbidities (e.g., post-neurosurgical management, *n* = 2, 10%). Of the 12 children with ongoing seizures post-PRES, 11 had complex underlying co-morbidities, for example previous bone-marrow or organ transplant. Three children (15%) experienced clinical evidence of PRES recurrence. Most children with recurrent PRES were changed to a different immunomodulating medication after their first PRES episode and experienced their second episode whilst on the alternate medication (e.g., Ciclosporin followed by Tacrolimus). Of those children that remained on long-term ASMs, 65% (*n* = 13) had a repeat EEG following PRES resolution. Repeat EEG showed a normal EEG in 4 children, no change in 3, and ongoing abnormalities in 6 children. The remainder did not undergo repeat EEG primarily due to transfers of care or relocation outside the UK.

### Neuroimaging findings

**Figure 4:**
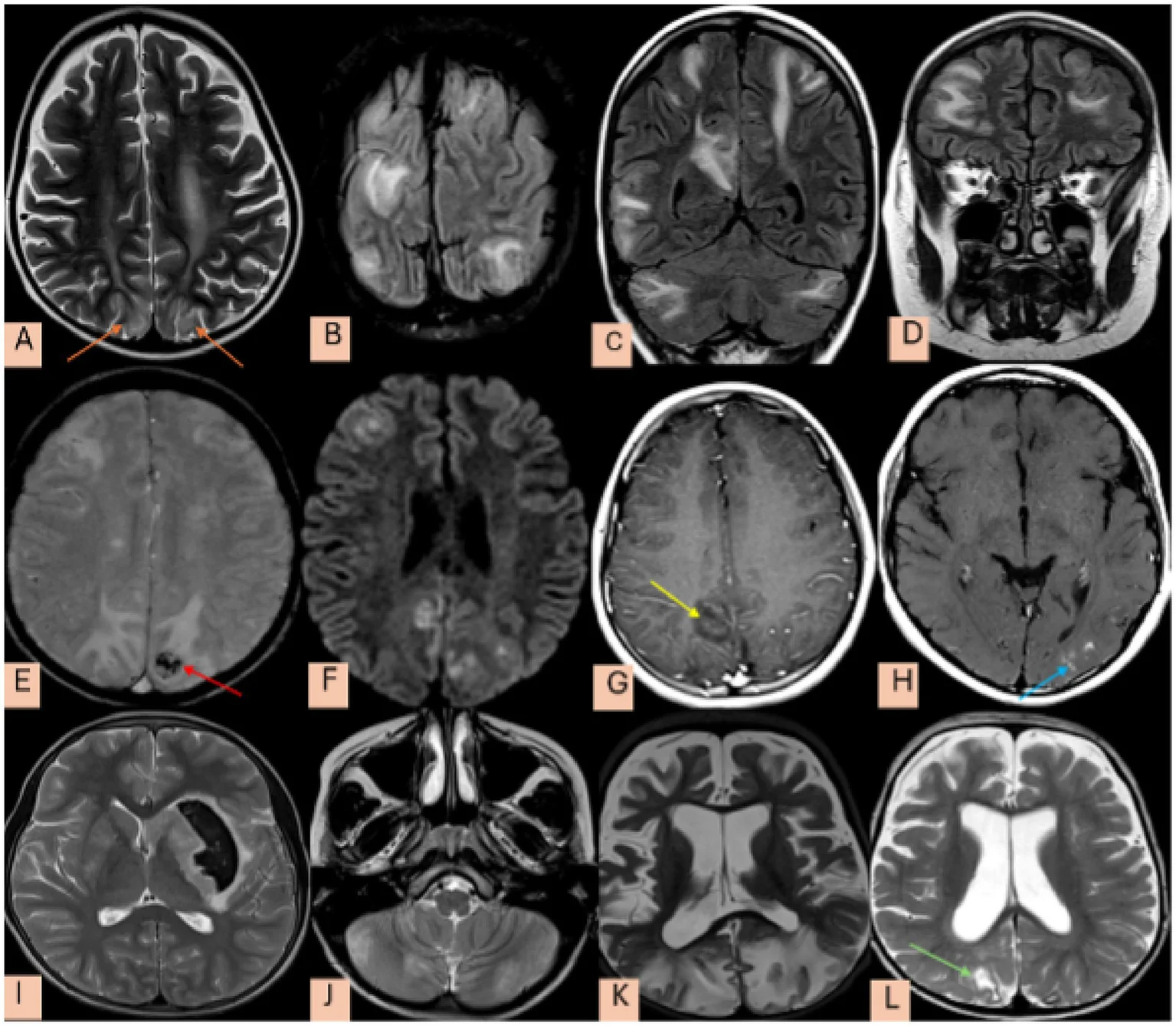
Examples of MRI findings in various paediatric cases. **(A,B)** Axial T2-weighted imaging (T2WI) and coronal Fluid-Attenuated Inversion Recovery (FLAIR) image showing bilateral posterior parietal occipital lesions (orange arrows) as well as cortical, and subcortical lesions, in keeping with a neuroradiological pattern typical of PRES. **(C,D)** Atypical lobar pattern of PRES showing multi-lobar and cerebellar involvement. This may be seen in atypical PRES imaging. **(E–H)** Atypical signal characteristics in four different patients with haemorrhage in left occipital region (**E**, red arrow), scattered intra-lesional foci of diffusion restriction **(F)**, intra-lesional parenchymal haemorrhage (**G**, yellow arrow) and leptomeningeal enhancement (**H**, blue arrow) in right posterior parietal and left occipital lesions. **(I,J)** Axial T2WI showing left ganglio-capsular bleed. Of note, there are patchy signal changes in right basal ganglia (I) and both cerebellar hemispheres **(J)**. **(K,L)** Axial T2Wis showing bilateral symmetric parieto-occipital lesions in panel **(K)**, leaving behind a gliotic focus (green arrow) in panel **(L)** on follow-up imaging in the same patient one year later. This is an example of persistent focal gliosis on follow-up imaging.

Neuroimaging was performed in all patients. Detailed neuroimaging findings are reported for the sixty-four children whose imaging was available and suitable for retrospective neuroradiological review (61 MRI and 3 CT). When considering the location of radiological patterns of involvement, 24 out of 64 children (37.5%) had the typical parieto-occipital pattern of involvement. Forty children (62.5%) had atypical patterns of involvement with frontal sulcal, other lobar, cerebellar and brainstem involvement constituting atypical patterns. One case had isolated basal ganglia involvement and one case had exclusive cerebellar involvement. Signal characteristics of radiological patterns were also considered. Thirteen children had lesions which showed diffusion restriction, nine cases had lesions with blooming in SWI images indicative of haemorrhages, five of them showed parenchymal enhancement and four showed focal leptomeningeal enhancement within the vicinity of the lesions (Figure 4). No overlap of haemorrhage and enhancement was noted in any of the patients.

#### Follow-up imaging

Follow-up imaging was obtained in 41 patients out of which five showed focal gliosis (Figure 4L) in PRES-affected regions; all five had atypical PRES imaging features at presentation. None of the patients in our cohort showed radiological signs of recurrence.

### Clinical correlation of imaging findings

Neuroimaging findings were correlated to the clinical presentations of PRES in our cohort.

#### Diffusion restriction (DWI+)

Thirteen children had evidence of positive diffusion restriction on imaging. Two of these patients experienced seizures post-PRES and two had notable neurological symptoms such as weakness or behavioural changes on follow-up. Interestingly, 11 patients had no seizures documented. Four of the above patients were prescribed long-term ASMs, primarily due to seizure burden or underlying neurological vulnerability (e.g., prior brain insult, steroid use, known neurology involvement).

#### Microhaemorrhage (SWI+)

The SWI + group comprised of nine children, of which two experienced seizures and two (*n* = 2) had ongoing neurological symptoms (e.g., hemiplegia, prior neurological impairment). Four of these patients were placed on long-term ASM, with reasons including recurrence of seizures and pre-existing neurological conditions.

#### Parenchymal enhancement

Parenchymal enhancement was seen in five patients. This group showed mixed clinical outcomes. Only one patient had seizures post-PRES and one had ongoing neurological symptoms. Two patients were on long-term ASM, one for suspected 5-methyltetrahydrofolate deficiency with seizures.

#### Leptomeningeal enhancement

Four children had evidence of leptomeningeal enhancement. Most patients in this group did not experience seizures or neurological symptoms. However, two patients were on long-term ASM, including one with abnormal EEGs and clinical seizures. Four of five (80%) had no ongoing follow-up. Although rare, leptomeningeal enhancement was associated with at least one case of significant clinical burden.

#### Follow-up gliosis

Gliosis was the most consistent imaging marker of long-term morbidity. Five patients demonstrated evidence of gliosis on follow-up imaging. Two of the five (40%) patients experienced seizures and two had clear ongoing neurological symptoms. Four of these patients were placed on long-term ASM, with one having multiple PICU admissions.

### EEG findings

Electroencephalographic (EEG) recordings were available for seventy-four patients, with abnormal findings identified in sixty-seven (*n* = 67/74, 90.5%). The majority of EEG referral questions (81.1%) aimed to determine the presence of electrographic seizures, while approximately one quarter (25.7%) inquired specifically about EEG features consistent with PRES. Other referral indications included encephalopathy (24.4%) and status epilepticus (9.5%) (Table 1).

**Table 1:** Neurophysiology referral indications for patients with PRES.

Referral question	Number of patients
Seizures	60 (81.1%)
Status epilepticus	7 (9.5%)
PRES	19 (25.7%)
Encephalopathy	18 (24.2%)

**Figure 5:**
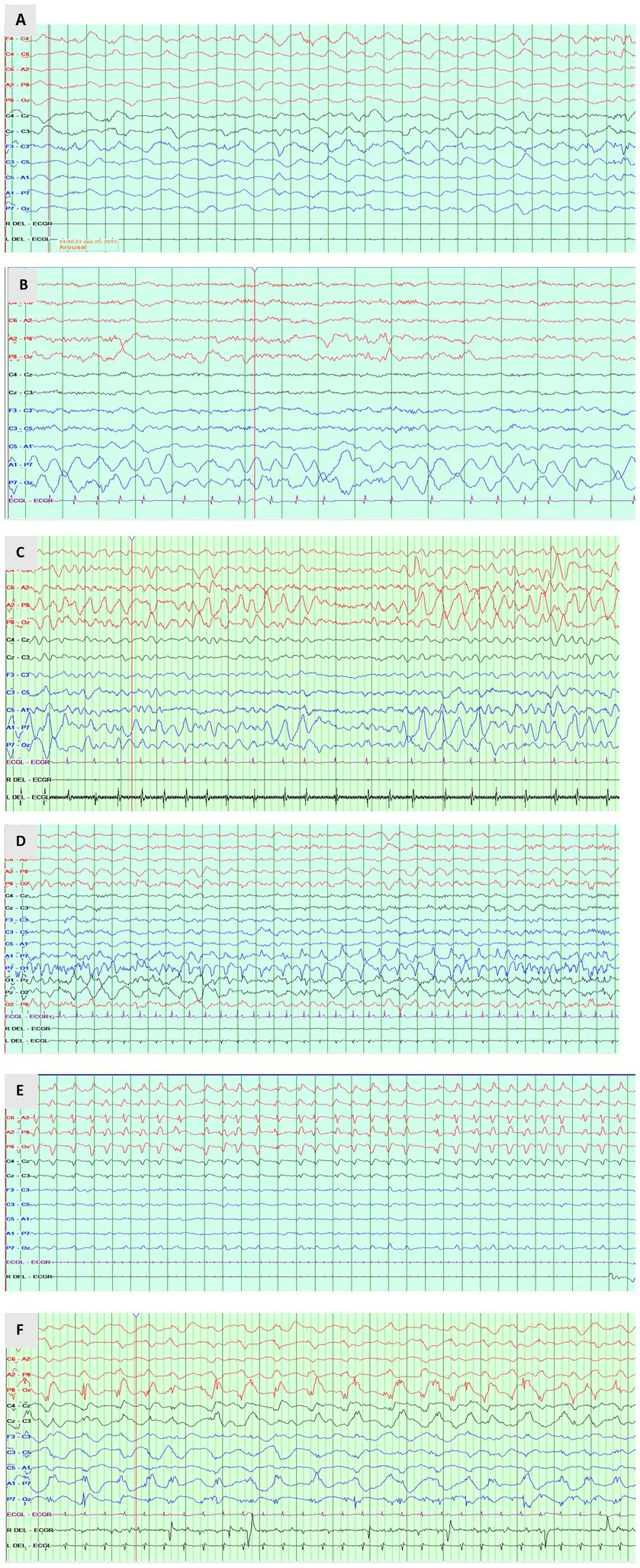
Examples of EEG findings. **(A)** Diffuse slowing in the EEG recording of a 10-year-old child indicating moderate generalised cerebral dysfunction. **(B)** Asymmetrical focal (left posterior) slowing. **(C)** Focal slowing (bilateral posterior). The EEG shows evidence of cerebral dysfunction most marked over the posterior regions bilaterally. **(D)** Left focal electrographic seizure (left posterior region). **(E)** Status epilepticus: Virtually continuous 1-2 Hz irregular sharp and slow wave discharges over the right hemisphere, particularly the right posterior region, with some spread to the left posterior region in keeping with focal status epilepticus involving the right hemisphere (The discharges resolved after intravenous Lorazepam, leaving a residual slow background). **(F)** Periodic lateralising epileptiform discharges (PLEDs) at around one per second which seem spikier over the right posterior region.

Significant ictal abnormalities, defined as focal electrographic seizures, were recorded in seven patients (9.5%), with two of these fulfilling criteria for non-convulsive status epilepticus (NCSE). In both NCSE cases, continuous or near-continuous rhythmic epileptiform discharges were observed over posterior regions. Posterior slowing was a frequent abnormality, seen in thirty-four (45.9%) EEGs, and was described as either symmetrical or asymmetrical with comparable frequency in both groups. Diffuse theta or delta slowing was reported in thirty-two (43.2%) patients, while periodic lateralized epileptiform discharges (PLEDs) were seen in three cases (4.1%). Only seven patients (9.5%) demonstrated a normal EEG (Figure 5).

### Clinical correlation of EEG findings

In this complex cohort, EEG abnormalities were common, with detectable changes in the vast majority (n = 67/74, 90.5%) of patients with available recordings. Multivariable logistic regression demonstrated a significant association between having an abnormal EEG and prolonged ASM use, adjusted for age group (likelihood ratio χ^2^ (8) = 22.17, p ≈ 0.0046; AIC = 68.83). Specifically, an abnormal EEG was independently associated with long-term ASM use (aOR = 4.03, 95% CI 0.86–18.89; *p* = 0.012). From the twenty children requiring long-term ASM, twelve (60%) exhibited focal slowing. Indeed, focal slowing was also an important predictor of long-term ASM use (likelihood ratio χ^2^ (8) = 20.30, p ≈ 0.009; AIC = 56.12), however only showed a trend towards higher odds when adjusted for underlying comorbidity and age (aOR = 1.49, 95% CI 0.28–7.88; *p* = 0.08). It is important to note the large confidence intervals of our findings, reflecting relatively small sub-group sample sizes for patients with normal EEGs and focal slowing. Other EEG features such as diffuse background slowing, focal slowing with a posterior emphasis, asymmetrical focal slowing, and PLEDs did not show significant links with post-PRES seizures or persistent neurological symptoms. Taken together, these data suggest that EEG abnormalities are commonly observed in paediatric PRES, and that any abnormality may predispose children to prolonged ASM requirements. Focal slowing was common in these children, suggesting a possible association that requires validation in larger prospective paediatric cohorts. Given the small subgroup sizes and heterogeneous comorbidities, this finding should be considered exploratory rather than evidence of independent prognostic value.

### Patient follow-up

Twenty-two children (28.6%) attended ongoing neurology outpatient follow-up after discharge. As PRES frequently occurred in children managed primarily by other services (district general hospitals, other specialist departments), children without dedicated neurology follow-up may have continued clinical follow-up under their primary specialty or local/home team. Of these patients, 10 experienced ongoing seizures, and twelve reported ongoing neurological symptoms such as balance loss, headaches, weakness, and vision changes. There was no associated mortality during the documented episode of PRES.

## Discussion

We present the largest UK paediatric PRES cohort to date, highlighting the range of complex comorbidities with which PRES may be associated with (8). The characteristics of our cohort are consistent with those reported in other large international, disease-specific cohorts, for example haemato-oncological conditions, and post-organ or haematopoetic cell transplantation (16, 17). Although rare, PRES can occur in previous well children, as observed in only two cases within our cohort. Consequently, our cohort is not representative of the wider paediatric population. Additionally, we report single-centre observations and practice, which may not reflect UK-wide trends.

PRES most commonly presented with new-onset seizures in our patients, consistent with existing literature (18). Hypertension was present in only 50 % of children, supporting previous literature reviews which suggest that hypertension is a less frequent presenting feature in paediatric patients compared to adults (9). This finding further supports the concept of PRES as a multifactorial syndrome, in which hypertension is not an obligatory feature. Hemida et al. (1) highlights several non-hypertensive triggers, including renal dysfunction, autoimmune and inflammatory disease, infection, and exposure to immunosuppressive or cytotoxic therapies. This is particularly relevant to our cohort, in which renal and immunological conditions were common, and many children were receiving immunomodulatory medication, similar to published international cohorts (19). Current evidence suggests that the pathophysiology of PRES may involve several interrelated mechanisms, including endothelial dysfunction, disruption of the blood–brain barrier, and impaired cerebral autoregulation, which may collectively contribute to the development of vasogenic oedema in response to these diverse triggers (1). Therefore, new onset seizures, irrespective of the presence of hypertension, should raise clinical suspicion for PRES, particularly in children with complex comorbidities. Furthermore, previous work comparing children younger than six years with older children has suggested that younger patients may experience more severe disease (17, 20). Presentation and disease severity, including ICU admission rates, were not influenced by children’s age in our, but also in other published cohorts (21). Reassuringly, there was no PRES-associated mortality in our cohort, and most children achieved full clinical recovery, which is in keeping with the existing consensus that PRES is predominantly a reversible and self-limited condition (5).

MRI remains the imaging modality of choice in suspected PRES, and should be interpreted alongside the clinical presentation, as atypical imaging patterns are increasingly recognised in children. Radiological abnormalities manifest as vasogenic oedema in parieto-occipital regions, classically bilateral and symmetric, without restricted diffusion or haemorrhage (22). However, atypical features are described, including involvement of the frontal and temporal lobes, cerebellum, basal ganglia, and brainstem (23). Advanced MRI sequences can provide prognostic insights; restricted diffusion has been linked to cytotoxic oedema and less favourable outcomes, while microhaemorrhages on susceptibility-weighted imaging (SWI) and post-contrast parenchymal or leptomeningeal enhancement have been associated with more severe presentations and prolonged neurological sequelae (24). Follow-up imaging typically shows resolution, but persistent gliosis has been highlighted as a marker of long-term morbidity (25). In our cohort, atypical radiological features were more common than those typical of PRES, a finding which has also been reported in the existing literature (18). Children with atypical features appeared more likely to require long-term anti-epileptic medication and have ongoing neurological symptoms, mirroring observations from other paediatric series (6, 26). However, statistical power was limited due to small subgroup sample sizes.

Electroencephalography (EEG) represents a further important diagnostic modality; however, its findings in paediatric PRES, and potential prognostic significance remain poorly characterised. Previous studies have highlighted heterogenous neurophysiological findings, including both diffuse background and focal slowing (27, 28), reinforcing that EEG abnormalities are neither a defining feature nor a prerequisite for diagnosing (29). In our cohort, most children demonstrated heterogeneous EEG abnormalities, and the presence of any EEG abnormality was associated with a greater likelihood of requiring long-term ASM. Interestingly, there was a non-significant trend towards an increased likelihood of long-term ASM use among children with focal slowing. Although this finding should be interpreted cautiously and considered hypothesis-generating, focal slowing may reflect persistent or localised cerebral dysfunction (30). Larger prospective, multicentre cohorts are required to determine whether this finding has true prognostic significance.

Furthermore, PRES management requires a multidisciplinary approach, with current evidence supporting predominantly supportive measures, including removing potential triggers, treating hypertension and managing acute seizures (5). There is limited evidence to guide the choice of ASM in paediatric PRES (9, 20, 31). In our cohort, levetiracetam was the most commonly used ASM, reflecting institutional prescribing practice and evolving prescribing patterns over the 20-year study period rather than evidence of superiority (32). Existing literature has proposed the use of non-enzyme inducing ASMs, such as sodium valproate or clonazepam, and has cautioned against the use of enzyme-inducing agents, such as phenytoin, because of potential interactions with chemotherapeutic and immunosuppressive agents^33,10.^ Importantly, Levetiracetam does not induce hepatic enzymes and has favourable pharmacokinetic properties, which may be particularly advantageous in children receiving chemotherapy or immunosuppressive therapies (32).

Most children only required short-term ASM therapy (<6 months), although twelve children required prolonged ASM therapy (>6 months). This reflects existing evidence of more frequent longer term complications in paediatric PRES compared to adult populations (9). Children requiring prolonged treatment generally experienced ongoing seizures or persistent neurological symptoms. Prolonged ASM use was also more common in children with complex comorbidities. In particular, children undergoing bone marrow or solid organ transplantation also had longer treatment durations throughout periods of immunosuppression, potentially reflecting increased central nervous system vulnerability (31). Overall, the variation in management observed in our cohort reflects the limited evidence available to guide paediatric PRES treatment and has helped inform the development of a more consistent local PRES pathway. The variation in follow-up in our cohort also highlights the lack of a standardised follow-up pathway for affected children. Overall, the differences in management observed in our cohort, and across the existing literature reflects the limited evidence base guiding current paediatric PRES management. Our findings have informed the development of a more consistent local management pathway, while larger multicentre consortia are needed to establish standardised national and international guidelines.

Antihypertensive therapy is another important treatment pillar. In our cohort, Amlodipine was most frequently prescribed. This reflects UK clinical practice, in which oral calcium channel blockers offer a well-tolerated route for blood pressure control (33). It is important to note that there are no specific evidence-based guidelines for blood pressure management in paediatric PRES. The heterogeneity in antihypertensive choice is evident across published literature, but most important is a gradual reduction in blood pressure by 20–25% within the first hours, minimising the risk of cerebral hypoperfusion without precipitating further neurological injury (34, 35).

It is important to acknowledge the limitations of our study. First, the retrospective, single-centre design is subject to variability in clinical documentation, follow-up, and management over the 20-year study period. Changes in diagnostic awareness and clinical practice, including greater recognition of PRES and its atypical presentations in more recent years, may have influenced case ascertainment, while advances in MRI technology and changing ASM prescribing practices may have affected observed imaging and treatment patterns. However, sensitivity analysis comparing diagnoses before and after 2015 showed no significant differences in investigation findings, ASM duration and PICU admissions (Table S1). Clinical management, including antihypertensive therapy, neuroimaging follow-up, EEG utilisation, and ASM duration, was not protocolised but reflected real-world clinical practice, which may have introduced treatment variability. Follow-up was also incomplete, reflecting the retrospective design and variation in clinical pathways within the UK National Health Service (NHS). This may have introduced a degree of ascertainment bias, with persistent or delayed neurological sequelae under-recognised in children without structured neurological follow-up. Secondly, historical imaging was unavailable for 13 children, and excluded from imaging analysis, because of changes in electronic and radiological archiving systems. Post-hoc analysis revealed that eight of these children subsequently died, predominantly from their underlying disease, raising the possibility of selection bias. However, this should be considered in the context of the substantial medical complexity of the overall cohort, in which 79.2% of patients had chronic comorbidities and 18.2% died due to their underlying disease. Patients excluded from imaging analysis therefore reflect the medically complex population from which our cohort was derived, rather than differences in PRES severity alone. Nevertheless, selection bias cannot be excluded, and conclusions regarding imaging findings should be limited to the neuroradiologically reviewed subgroup. Thirdly, although this represents the largest UK paediatric PRES cohort to date, the rarity of PRES, heterogenous clinical presentations, and underlying diagnoses, limited statistical power, particularly for subgroup analyses. Findings from small subgroups should therefore be interpreted cautiously. Given the observational design of this study, imaging findings should be interpreted as descriptive rather than demonstrating statistical associations. Finally, as a tertiary referral centre, our cohort included an over-representation of children with complex comorbidities, which limits generalisability to general paediatric populations.

Our cohort reinforces and advances our current understanding of paediatric PRES presentation and management. Our findings identify exploratory associations between EEG and atypical MRI patterns and longer-term outcomes; however, their prognostic significance requires validation in larger prospective cohorts. Future studies should build upon these observations and lead to the development of evidence-based paediatric guidelines, which remain lacking despite the increasing recognition of this entity.

## Data Availability

The original contributions presented in the study are included in the article/supplementary material, further inquiries can be directed to the corresponding author.
